# Effect of fluoride varnish on glass ionomer microhardness changes in endogenous acid erosion challenge

**DOI:** 10.1080/26415275.2021.1880907

**Published:** 2021-02-10

**Authors:** Fatemeh Moharramkhani, Ladan Ranjbar Omrani, Mahdi Abbasi, Mohammad Javad Kharrazifard, Elham Ahmadi

**Affiliations:** a Dental Research Center, Dentistry Research Institute, Tehran University of Medical Sciences, Tehran, Iran; b Department of Operative Dentistry, School of Dentistry, Tehran University of Medical Sciences, Tehran, Iran

**Keywords:** Hardness tests, glass ionomer, gastric acid

## Abstract

**Objective:**

This study aimed to assess the effect of fluoride varnish on glass ionomer microhardness changes after endogenous acid erosion challenge.

**Methodology:**

In this study, 40 conventional glass ionomer (CGI; Fuji IX) and 40 resin-modified glass ionomer (RMGI; Fuji IILC) discs were fabricated and divided into 4 subgroups (*n* = 10) for immersion in synthetic gastric acid or saliva for 27 h with/without fluoride varnish application. The surface microhardness was measured at baseline and after immersion, and the change in microhardness was calculated. Data were analyzed using analysis of variance and T-test.

**Results:**

A reduction in microhardness was noted in all subgroups following immersion. The lowest change in microhardness of both CGI and RMGI occurred in artificial saliva. In CGI groups, the highest reduction in microhardness occurred in synthetic gastric acid with fluoride varnish application, and the reduction was significantly different from that of the CGI group with fluoride varnish application (*p* value = .01). In RMGI groups, the highest reduction in microhardness was noted in synthetic gastric acid without fluoride varnish application, and the reduction was significantly different from that of the other groups (*p* value < .05).

**Conclusions:**

Exposure to synthetic gastric acid caused a significant reduction in microhardness of RMGI. Varnish application significantly decreased the acid susceptibility of RMGI, but not that of CGI.

## Introduction

Dental erosion occurs as the result of exposure of tooth structure to acid attacks without the involvement of microorganisms [1,2]. The reported prevalence of dental erosion varies from 27 to 83% [3]. Dental erosion is caused by the interaction of chemical, biological and behavioral factors [4,5]. Chemical factors, such as the dietary (extrinsic) and gastric (intrinsic) acids prompt softening and degradation of tooth structure [2,6,7]. The erosive property of gastric acid is significantly higher than that of acids present in the nutritional regimen. The pH of gastric acid is very low (<2) and below the critical pH for enamel demineralization (5.5) [8].

Erosive lesions may become so extensive as to require restoration to control dentin hypersensitivity, decrease the risk of pulp exposure, protect the residual tooth structure, enhance the esthetic appearance and reconstruct the vertical dimension [9]. Direct and indirect restorative procedures are recommended for such teeth [10–12]. Conventional glass ionomer (CGI) or resin-modified glass ionomer (RMGI) cements and composite resins are the most commonly applied direct restorative materials for this purpose [9].

The fluoride release potential, chemical bonding to tooth structure, and having a coefficient of thermal expansion close to that of tooth structure are some of the favorable properties of glass ionomer cements [9,13].

Viana et al. [9] stated that glass ionomer-based materials caused the lowest erosive damage of the adjacent enamel. Turssi et al. [14] showed that fluoride release of glass ionomer restorations had an inhibitory effect on root dentin of teeth subjected to erosive acidic challenge. However, these findings were not confirmed by Francisconi et al. [15] and Rios et al. [11] who reported no improvement in enamel resistance adjacent to glass ionomer restorations to acid attacks.

In addition, use of fluoride-containing products has been suggested to inhibit, manage, and treat tooth demineralization that takes place in the process of dental erosion [1,2,9]. The availability and accessibility of minerals to reverse the process of demineralization is a concern for patients experiencing repeated erosive challenges, and materials that can be bonded to the tooth surface, such as sealants, varnishes and gels may be more effective for such patients [1,4]. Products, such as fluoride varnish with high fluoride content and long durability on the tooth surface (12 h) are often the first treatment option against tooth erosion/abrasion challenges due to the deposition of CaF_2_ in large amounts [1,4,16]. The effects of erosive challenges on restorative materials are different from their effects on the tooth structure [9]. Erosion can compromise the mechanical behavior of restorative materials, decrease their hardness, increase their roughness and decrease their longevity and clinical service [1,9]. Pretreatment of restoration surfaces with fluoride varnish has been suggested to enhance their resistance to erosion [1,17].

Considering the scarcity of evidence regarding the preventive effects of fluoride varnish on erosion of glass ionomers, this experimental study aimed to assess the preventive effects of fluoride varnish on glass ionomer microhardness changes in endogenous acid erosion challenge.

## Methodology

This *in vitro*, experimental study was conducted on Fuji IILC (GC Corp., Tokyo, Japan) and Fuji IX (GC Corp., Tokyo, Japan). Sample size was calculated to be 10 in each subgroup using advanced one-way ANOVA power analysis, considering alpha = 0.05, beta = 0.2, effect size of 0.45 and standard deviation of 2.6 [18].

### Preparation of storage media

The gastric acid was synthesized by dissolving 2.0 g of sodium chloride in 3.2 g of pepsin and 7.0 ml of hydrochloric acid; water was added to reach the final volume to 1000 ml. The pH of the solution was measured by a pH meter to be 1.14 [19].

Next, 1.5 mmol/L Ca, 0.9 mmol/L P, 150 mmol/L KCl, and 0.05 mg F/mL in 0.1 mol/l Tris buffer were mixed to prepare artificial saliva with a pH of 7 [20].

### Preparation of samples

Forty samples were fabricated from Fuji II LC RMGI, and 40 samples were fabricated from each type of glass ionomers (Fuji IILC and Fuji IX) measuring 2 × 7 mm using a metal mold according to the manufacturers’ instructions.

During the setting process (light-curing or chemical setting), a glass slide was placed over the molds in order to leak-out the excess cement and obtain a smooth surface. The RMGI was light-cured using a LED curing unit (Guilin Woodpecker Medical Instrument Co., Guilin, China) with a light intensity of 1000 mW/cm^2^ through a glass slide from both sides, each for 30 s. The Fuji IX samples were removed from the mold after 10 min. Both the CGI and RMGI samples were polished with 1000- and 1200-grit abrasive papers with 10 strokes on each side after 24 h of incubation at the temperature of 37 °C. The samples were then mounted in wax sheets.

The samples in each of the RMGI and CGI groups were divided into four subgroups. Table 1 summarizes the subgroup classification and the procedural steps.

**Table 1.** Classification of subgroups.

Type of glass ionomer/subgroups		Surface treatments		Procedural steps
		Acid	Varnish	
RMGI	RMGIa	+	−	a: Samples in both RMGIa and CGIa subgroups were immersed in synthetic gastric acid and incubated at 37 °C for 27 h. va: Samples in RMGIva and CGIva subgroups were treated with Fluor Protector fluoride varnish (Ivoclar Vivadent, Schaan, Lichtenstein) and were then immersed in synthetic gastric acid and incubated at 37 °C for 27 h. c: Samples in both RMGIc and CGIc subgroups were immersed in artificial saliva and incubated at 37 °C for 27 h (negative control). v: Samples in both RMGIv and CGIv subgroups were treated with Fluor Protector fluoride varnish and were then immersed in artificial saliva and incubated at 37 °C for 27 h.
	RMGIva	+	+	
	RMGIv	−	+	
	RMGIc	−	-	
CGI	CGIa	+	-	
	CGIva	+	+	
	CGIv	−	+	
	CGIc	−	-	

For surface treatment with Fluor Protector fluoride varnish, the varnish was applied on polished surfaces with a microbrush and allowed 60 s to dry. Prior to immersion, the baseline surface microhardness of all samples was measured in all subgroups using Vickers hardness tester (Bareiss, Baiersbronn, Germany). A square-based diamond pyramid indenter with an apical angle of 136° applied 50 N load for 10 s. The test was performed in triplicate for each sample, and the mean of the three values was calculated and recorded as the microhardness of the respective sample. The samples were then incubated as explained above (37 °C for 27 h). After incubation, they were rinsed with distilled water and air-dried. Microhardness of the samples was then measured again. The change in microhardness was calculated:

### Statistical analysis

Three-way analysis of variance was used to assess the effect of immersion medium (synthetic gastric acid or artificial saliva), application of Fluor Protector fluoride varnish (yes/no), and type of glass ionomer (Fuji IX or Fuji II LC) on the change in microhardness of glass ionomers. The interaction effect of these factors was significant (*p* value < .05). Thus, the effect of each parameter on the change in microhardness of glass ionomers was separately analyzed using one-way ANOVA.

## Results

Table 2 presents the mean change in microhardness of RMGI and CGI samples in the eight subgroups. A reduction in microhardness was noted in all subgroups after immersion in gastric acid or artificial saliva.

**Table 2.** Mean and SD change in microhardness of subgroups (*n* = 10) and SD.

Subgroups Glass ionomer	c	a	v	va
RMGI	14.12 ± 15.51^cA^	66.01 ± 16.64^aA^	15.14 ± 5.75^cB^	35.26 ± 20.99^bA^
CGI	16.98 ± 20.41^bA^	33.88 ± 34.67^abB^	30.94 ± 17.25^bA^	51.58 ± 16.87^aA^

SD: Standard deviation

Groups denoted by the same decimal letter in the same row represent no significant difference, and groups denoted by the same capital letters in the same column represent no significant difference. (*p* > .05).

Some samples in RMGIa (*n* = 2), CGIa (*n* = 6) and CGIva (*n* = 5) subgroups were severely destroyed in acid such that their secondary surface microhardness was not measurable. Thus, their secondary microhardness was considered zero.

Regarding the effect of type of glass ionomer, RMGIa showed a significantly higher change in microhardness than did CGIa (*p* = .02), and CGIv showed a significantly higher change in microhardness than did RMGIv (*p* = .01).

Regarding the effect of immersion medium, a significant difference was noted in the change in microhardness of RMGIa and RMGIc (*p* < .001), RMGIva and RMGIv (*p* = .01) and CGIva and CGIv (*p* = .01).

Regarding the effect of fluoride varnish application, significant differences were noted in the change in microhardness of RMGIa and RMGIva (*p* = .002).

## Discussion

This study assessed the preventive effect of fluoride varnish on glass ionomer microhardness changes in endogenous acid erosion challenge.

Previous studies on the protective effect of fluoride on erosion of glass ionomers following exposure to exogenous acids have reported controversial results [17,21] due to differences in erosion cycles and protocols, pH of acids, type of acids, duration of erosion and different types of fluoride products used. No study was found on the protective effect of fluoride on wear of glass ionomers following exposure to gastric acid. However, Cengiz et al. [19] assessed the effect of gastric acid on laboratory composites, Zaki et al. [22] assessed the effect of gastric acid on different glass ionomers and composites and Sulaiman et al. [10] assessed its effect on monolithic zirconia. The aforementioned studies used hydrochloric acid to simulate gastric acid with a pH range of 1.2 [23] to 3.8 [22]. In addition to hydrochloric acid, gastric acid contains different enzymes, such as pepsin with proteolytic properties that can degrade the collagen [24]. The results of studies regarding the aggravating effect of pepsin on dentin erosion are controversial [25,26]. A previous study showed significantly higher amounts of pepsin, trypsin and amylase in the saliva of patients with chemical erosion caused by endogenous acids, compared with normal individuals [26].

In this study, hydrochloric acid was used along with pepsin to prepare a solution simulating gastric acid according to the protocol described by Cengiz et al. [19]. Duration of exposure was set at 27 h, corresponding to 9 years of clinical acid exposure (30 s of acid exposure for averagely 7 times a week) [22]. However, continuous immersion in the saliva for 27 h is different from the clinical conditions, which can be considered as a limitation of this study and could have resulted in exaggeration of the situation. The conditions are probably better in the clinical situation due to the presence of saliva and its buffering capacity, and repeated acid exposures.

Although the pH of the acidic solution and duration of immersion in acid in the study by Zaki et al. [22] were different from this study, they reported minimum microhardness in RMGI group following 6 h of immersion, corresponding to 2 years of acid exposure in the clinical setting. They reported that the microhardness of CGI and RMGI samples following 13 h of acid exposure (corresponding to 4 years of clinical acid exposure) had significant difference with that of the control subgroup. In this study, the results after 27 h of acid exposure were in line to their findings after 6 h and 13 h of exposure.

It seems that the resin matrix of RMGI has a higher resistance to dissolution than CGI. But, HEMA has hydrophilic nature and two phenomena might happen. The first one is that HEMA acts as a hydrogel when exposed to water, and the second one is its separation due to the continuation of acid-base reactions. The matrix becomes hydrophobic, and HEMA is separated from other phases [8].

In this study, exposure of RMGI to gastric acid significantly decreased its microhardness. However, gastric acid did not have a significant effect on the CGI samples. The reactive sites are evenly distributed in CGIs (unlike RMGIs); thus, the effect of acid on all areas would be the same upon acid attack. However, in RMGIs, the weakest area around glass particles is more severely affected by acid, leading to a selective destruction pattern, which further degrades the surface of RMGIs. This fact along with the presence of HEMA may explain the highest change in microhardness in the RMGIa subgroup. On the other hand, evidence shows that the equilibrium of acid-base reactions and the maximum polymerization of glass ionomers require 7 d to occur; thus, incubation at 37 °C for 7 d is often recommended [15]. However, we did not adhere to this protocol in our study, which might have further complicated the situation.

Contrary to our study, Yu et al. [17] showed that application of amine fluoride in a simulated erosive environment significantly prevented the surface degradation of CGI. However, they used citric acid in their study. They hypothesized that formation of a fluoride-rich layer on the surface of glass ionomers and compomers may reinforce their surface against acid attacks. According to Schlueter et al. [25] presence of pepsin in gastric acid had no significant effect on demineralization of tooth structure but adversely affected the efficacy of fluoride in prevention of erosion. This explains the inefficacy of fluoride varnish in CGI Group in acidic environment. In CGIva subgroup, varnish application decreased the acid susceptibility of CGI. According to the authors’ opinion, the different composition of CGI and RMGI may be responsible for the different behavior of varnish application in acidic condition. On the other hand, Fluor Protector contains 0.9% difluorsilane in a polyurethane varnish base with ethyl acetate and isoamylpropionate solvents, and this composition may have undesirable effects on the surface of CGIs.

The significant difference between the CGIv and RMGIv subgroups may further corroborate this hypothesis.

Regarding the immersion medium, minimum change was noted in RMGIc and CGIc subgroups. Also, the difference in the change in microhardness was significant between RMGIa and RMGIc, RMGIva and RMGIv, and also between CGIva and CGIv subgroups. In other words, the acidic medium caused greater change in microhardness than artificial saliva.

In the CGI samples, application of fluoride varnish (irrespective of immersion medium) decreased the microhardness; however, the difference between the CGIc and CGIv was not significant. As mentioned earlier, gastric acid contains pepsin, which prevents the effect of fluoride. Thus, the change in microhardness in CGIva subgroup was higher than that in the CGIv subgroup. On the other hand, the acidic medium has an adverse effect on hydrogel areas around glass particles, compared with saliva.

This study had some limitations. We did not consider the buffering capacity of the saliva, and exposed the samples to acid continuously for 27 h, which is different from the clinical condition. Thus, our findings cannot be generalized to the clinical setting. Moreover, in the clinical setting, aside from the endogenous acid attacks, nutritional regimen of patients may include exogenous acids, which affect the process of erosive attacks. On the other hand, oral hygiene in the clinical setting may aggravate the erosion. Absence of acquired pellicle (which has a protective effect on tooth and restorative materials) can also result in a different behavior *in vitro*, compared with *in vivo*. Future studies are required to assess the effect of fluoride varnish on microhardness of other tooth-colored restorative materials following exposure to endogenous and exogenous acids. Also, the effect of topical fluoride solutions on improvement of physical properties and strength of different restorative materials particularly glass ionomers should be investigated in future studies.

Exposure to synthetic gastric acid caused a significant reduction in microhardness of RMGI samples. Application of fluoride varnish over Fuji II LC RMGI decreased the change in microhardness in acidic media, while it increased the reduction in microhardness of Fuji IX CGI; however, the difference was not significant. It means that varnish application only had a preventive effect on RMGI against the endogenous acid erosion challenge.

## Data Availability

The data used to support the findings of this study are available from the corresponding author upon request.
